# Degradation of Tetracyclines in Pig Manure by Composting with Rice Straw

**DOI:** 10.3390/ijerph13030254

**Published:** 2016-02-24

**Authors:** Rushan Chai, Lidong Huang, Lingling Li, Gerty Gielen, Hailong Wang, Yongsong Zhang

**Affiliations:** 1 Ministry of Education Key Laboratory of Environmental Remediation and Ecosystem Health, College of Environmental and Resource Sciences, Zhejiang University, Hangzhou 310058, China; rschai@ahau.edu.cn (R.C.); lingjiaren@gmail.com (L.L.); 2 School of Resources and Environment, Anhui Agricultural University, Hefei 230036, China; 3 Jiangsu Provincial Key Laboratory of Agricultural Meteorology, College of Applied Meteorology, Nanjing University of Information Science & Technology, Nanjing 210044, China; ldhuangnz@163.com; 4 Scion, Private Bag 3020, Rotorua 3046, New Zealand; gerty.gielen@scionresearch.com; 5 Key Laboratory of Soil Contamination Bioremediation of Zhejiang Province, School of Environmental and Resource Sciences, Zhejiang A & F University, Lin’an 311300, China; nzhailongwang@gmail.com

**Keywords:** pig manure, rice straw, composting, tetracyclines, degradation

## Abstract

A holistic approach was followed for utilizing tetracyclines (TCs)-contaminated pig manure, by composting this with rice straw in a greenhouse for CO_2_ fertilization and composted residue application. After composting, the composted residues can be applied to cropland as a supplemental source of synthetic fertilizers. The objective of this study was to determine the effect of pig manure-rice straw composting on the degradation of TCs in pig manure. The results showed that greenhouse composting significantly accelerated the degradation of TCs. Contents (150 mg·kg^−1^) of oxytetracycline (OTC), tetracycline (TC) and chlortetracycline (CTC) in the composting feedstock could be completely removed within 42 days for OTC and TC, and 14 days for CTC. However, in the control samples incubated at 25 °C in the dark, concentrations of OTC, TC and CTC only decreased 64.7%, 66.7% and 73.3%, respectively, after 49 days. The degradation rates of TCs in the composting feedstock were in the order of CTC > TC > OTC. During the composting process, CTC dissipated rapidly with the time required for 50% degradation (DT_50_) and 90% degradation (DT_90_) of 2.4 and 7.9 days, but OTC was more persistent with DT_50_ and DT_90_ values of 5.5 and 18.4 days. On the basis of the results obtained in this study, it could be concluded that pig manure-rice straw composting in a greenhouse can help to accelerate the degradation of TCs in pig manure and make composted residues safer for field application. This technology could be an acceptable practice for greenhouse farmers to utilize TCs-contaminated pig manure.

## 1. Introduction

With the booming of intensive livestock breeding, about 465 million tonnes of pig manure is generated annually in China [1]. However, most pig farms in China do not have facilities for the treatment and disposal of manure and wastewater [2]. The direct discharge of animal wastes contributes significantly to water quality deterioration and also results in a great loss of valuable nutrients. Furthermore, animal manure frequently contains residues of veterinary antibiotics [3,4,5]. In recent years, antibiotics are being widely used as feed additives and growth promoters in the intensive animal husbandry, which are only partially metabolized in the animal body before being excreted [6]. Tetracycline (TC), oxytetracycline (OTC) and chlortetracycline (CTC) are three typical and the most frequently used tetracycline antibiotics in animal husbandry [7]. In China, TC, CTC and OTC were the most prominent contaminants detected in pig manure samples, with maximum concentrations reaching 98, 139 and 354 mg·kg^−1^ DW, respectively [8]. With the application of antibiotic-contaminated manure, antibiotic residues were widely detected in soil, vegetables and groundwater, which undoubtedly has an impact on the safety of ecosystems and human health [9,10]. In Northern China, tetracyclines (TCs) were the most frequently detected antibiotics in manure-applied agricultural soils [11]. Moreover, the spread of antibiotic resistance gene following land application of animal manure has become an issue of considerable concern [12,13,14]. Therefore, it is essential to treat animal manure effectively before its application as fertilizer to minimize the potential risks from antibiotic residues.

In China, the rapid development of agriculture in recent years has resulted in an output of about 190 million t·yr^−1^ of rice straw [15]. Because there is no profit in recycling straw and leaving it on cropland will adversely affect growth of the next season’s crop, most farmers choose to burn the straw directly in the field [16]. In China, about 23% of rice straw is burnt in open field [15]. This burning of crop straw after harvest is a significant seasonal source of air pollution [17]. However, many farmers lack the technology and financial support for more environment-friendly ways of treating straw, such as burying straw deeply for fertilizing the soil or digestion for methane generation [16].

In nearly airtight greenhouses, CO_2_ concentrations are considerably lower in winter. This problem is exacerbated in the middle of the day when vegetables experience their maximum photosynthetic rates. In our investigation, it was discovered that daily CO_2_ concentrations in the control greenhouses decreased from 500 μmol·mol^−1^ at 08:00 am to 200 μmol·mol^−1^ at 12:00 noon [18]. For this reason, CO_2_ fertilization was strongly recommended in greenhouses in winter. In order to produce organic fertilizer from composting and also beneficially use the CO_2_ produced, a system was designed in which CO_2_ fertilization in a greenhouse was achieved by composting pig manure-rice straw inside the greenhouse. In our previous work, the dynamic changes of CO_2_ concentration in the composting greenhouses, and the positive effects of this technology on vegetable production and quality have been determined [18]. Elevated CO_2_ concentrations in greenhouses, fitted with composting units, not only increased vegetable yields by 87%–270%, but also improved the vegetable quality in terms of increased soluble sugars and vitamin C content, and decreased nitrate content [18]. However, further research on the degradation of TCs during the composting process is lacking. The major purpose of the present study was to investigate the behavior and fate of TCs during this composting process. The results could provide useful information for assessing the environmental safety of composted residues in agricultural application.

## 2. Materials and Methods

### 2.1. Chemicals and Composting Materials

Oxytetracycline dihydrate, tetracycline and chlortetracycline hydrochloride were purchased from Sigma Co. (St. Louis, MO, USA) and used without further purification. Their acid dissociation constant (p*K*a) values are shown in Table 1. All chemicals used in this experiment were reagent grade. Oxalic acid dihydrate, 95% ethanol and sodium chloride (NaCl) were sourced from Wako Pure Chemical Industries (Osaka, Japan). Acetonitrile and methanol were high-performance liquid chromatography (HPLC) grade and purchased from Merck (Darmstadt, Germany). Deionized water (18.1 MΩ) was used throughout the experiment. Tetracycline and chlortetracycline hydrochloride were stored at −20 °C. Oxytetracycline dihydrate and other reagents and solvents were stored in the dark at 25 °C. The characteristics of pig manure and rice straw used for experiments are shown in Table 2. Pig manure was collected from the farm where antibiotics were not used, and no TCs were detected in this manure.

### 2.2. TCs Degradation Experiments

**Figure 1:**
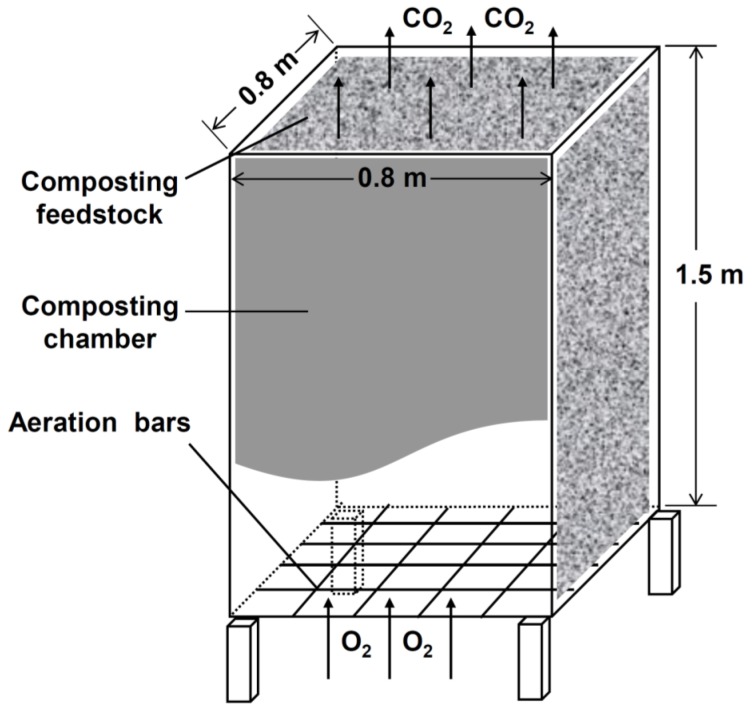
Schematic illustration of the pig manure-rice straw composting unit.

The composting experiment was conducted at an experimental farm of Zhejiang University. Three composting units were positioned inside a greenhouse (50 × 6 × 3 m). The composting unit contained a base fence (1 × 1 m) constructed from bamboo with a diameter of 5 cm (Figure 1). The base fence was elevated 20 cm from the ground to facilitate ventilation. A composting chamber (0.8 × 0.8 × 1.5 m) made from plastic sheeting was placed on top of the base fence. This chamber, open at the top and bottom, was propped up with four bamboo sticks. Optimum conditions for pig manure-rice straw composting in terms of CO_2_ production were a C/N ratio of 40 and water content of 70% [19]. In order to achieve the optimal C/N ratio, the feedstock was added continuously at a rate of 30 kg rice straw and 5 kg moist pig manure until the composting chamber was filled with about 300 kg rice straw and 50 kg pig manure. During the filling process, the feedstock was inoculated with a mixture of the fungi *Aspergillas niger* zj1, *Trichoderma viride* zj2 and *Panusconclmtw* zj3 by spraying enriched solution to accelerate composting for CO_2_ release.

For the TCs degradation experiments, rice straw was cut into 2 cm size pieces using scissors. The OTC, TC and CTC were dissolved in methanol each at a concentration of 1 g·L^−1^, one hour prior to the experiment. To avoid any potential effects of the solvent on the microbial activity of the composting feedstock, 4.5 mL of methanol containing TCs was first added to pig manure, placed in 100 mL plastic beakers, and then mixed with rice straw to achieve a separate 30 g dried sample of composting mixture using the method described by Brinch *et al.* [20]. Briefly, the sample was air-dried in a fume cupboard for approximately 5 h, and intermittently stirred with a glass rod. This resulted in final TCs concentrations of 150 mg·kg^−1^ each. Then, the water content of the samples was adjusted to 70%. Three plastic beakers with the composting samples were each put into nylon bags (15 × 10 cm, mesh size: 0.15 mm), tied tightly and buried into three composting units at a depth of 60 cm. Three plastic beakers with the control samples were covered with Parafilm, leaving four holes for ventilation, and incubated at 25 °C in the dark for 49 days. The water content of the control samples was checked weekly by weighing and kept constant by adding deionized water. The composting and control samples were simultaneously extracted to determine TCs at day 0, 7, 14, 21, 28, 35, 42 and 49, respectively. Three replicates per treatment were collected on each sampling date. The temperature of the central part of the composting pile was monitored per hour using an auto-recording thermometer (ZDR-21, Hangzhou Zeda Instruments Co., Ltd.: Hangzhou, China). The pH was determined in suspensions of 1: 10 (W/V) sample/2 M KCl using a pH meter (PB-10, Sartorius: Goettingen, Germany). The organic carbon (OC) and total nitrogen (TN) contents of the composting feedstock were measured according to the methods described in Bao [21].

### 2.3. Extraction and HPLC Analysis of TCs

The TCs were extracted from the subsamples and analyzed using an optimized method described in our previous study [22]. Briefly, subsamples (2 g) were placed in 10 mL centrifuge tubes and extracted three times with extraction buffer (4 mL, 0.5 M oxalic acid – 1 M NaCl – ethanol = 25:25:50, *v*/*v*/*v*) by vortexing for 10 s followed by sonication for 15 min. After each extraction, the extracts were centrifuged at 2500 r·min^−1^ for 10 min, and the supernatants were collected and centrifuged again at 3000 r·min^−1^ for 10 min, filtered through cellulose acetate membrane filters and analysed by HPLC. The HPLC analyses of the TCs were carried out using an 1100 series HPLC system (Agilent, Palo Alto, CA, USA) equipped with a UV detector, auto-sampler and a Cosmosil 5C18-AR-II column (4.6 mm I.D. × 250 mm, Waters, Milford, MA, USA) at ambient temperature (23 ± 1 °C). The mobile phase of 0.01 M oxalic acid-ACN-methanol (79:10.5:10.5, *v*/*v*/*v*) was pumped at a flow rate of 1.0 mL·min^−1^. The sample injection volume was 5 μL, and light absorption detected at 268 nm.

### 2.4. Data Analysis

Data analysis was performed by using Data Processing System (DPS) 2000 statistical software package (Zhejiang University, Hangzhou, China).

## 3. Results

### 3.1. Evolution of Temperature during Composting

**Figure 2:**
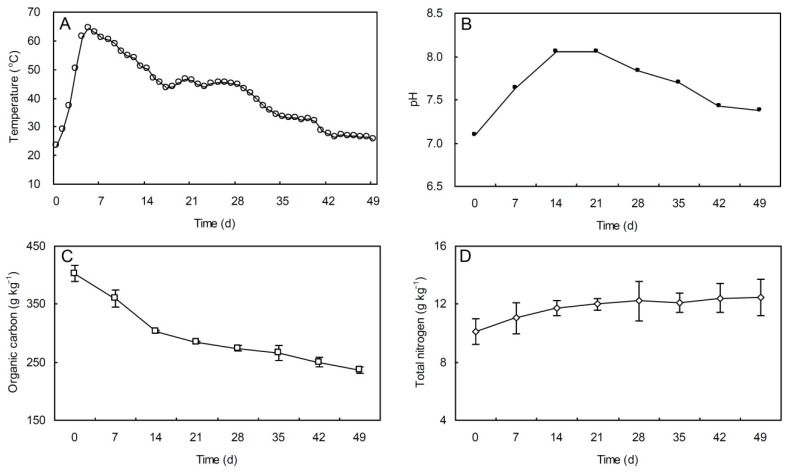
Evolution of temperature (**A**); pH (**B**); organic carbon (**C**) and total nitrogen (**D**) during pig manure-rice straw composting. Bars represent standard deviation.

In greenhouses, the pig manure-rice straw composting process is initiated by the microbiological decomposition of mixed organic materials. The temperature within the composting pile was monitored during the 49 days composting period (Figure 2). This showed that the evolution of the temperature within the composting pile went through three major phases: heating phase, thermophilic phase, and cooling phase. At the beginning of composting, the temperature continually increased and reached a maximum of 64.5 °C, as a consequence of the heat generated as a result of biodegradation of the composting feedstock. The composting pile achieved thermophilic temperature (>50 °C) on day 3 of the composting cycle. This thermophilic phase lasted for approximately 12 days, after which the cooling phase started when the temperature gradually decreased due to the depletion of compostable organic matter.

### 3.2. Evolution of pH, OC and TN During Composting

At the beginning of the composting process, the value of pH was 7.09. During the composting process, the microbial activities resulted in production of NH_3_ that increased the pH rapidly to a maximum value of 8.06 on day 14. After that, due to the volatilization or microbial assimilation of ammonical nitrogen, the pH gradually declined and reached a value of 7.38 at the end of composting. The release of CO_2_ might also be responsible for a decrease in the pH value [23]. During the composting process, the organic carbon content decreased from 403 to 236 g·kg^−1^, due to microbiological decomposition of organic matter and conversion of C to CO_2_. And the total nitrogen content of the composting feedstock increased from initial 10.1 to 12.5 g·kg^−1^ (Figure 2).

### 3.3. Degradation of TCs during Composting and Incubation

**Figure 3:**
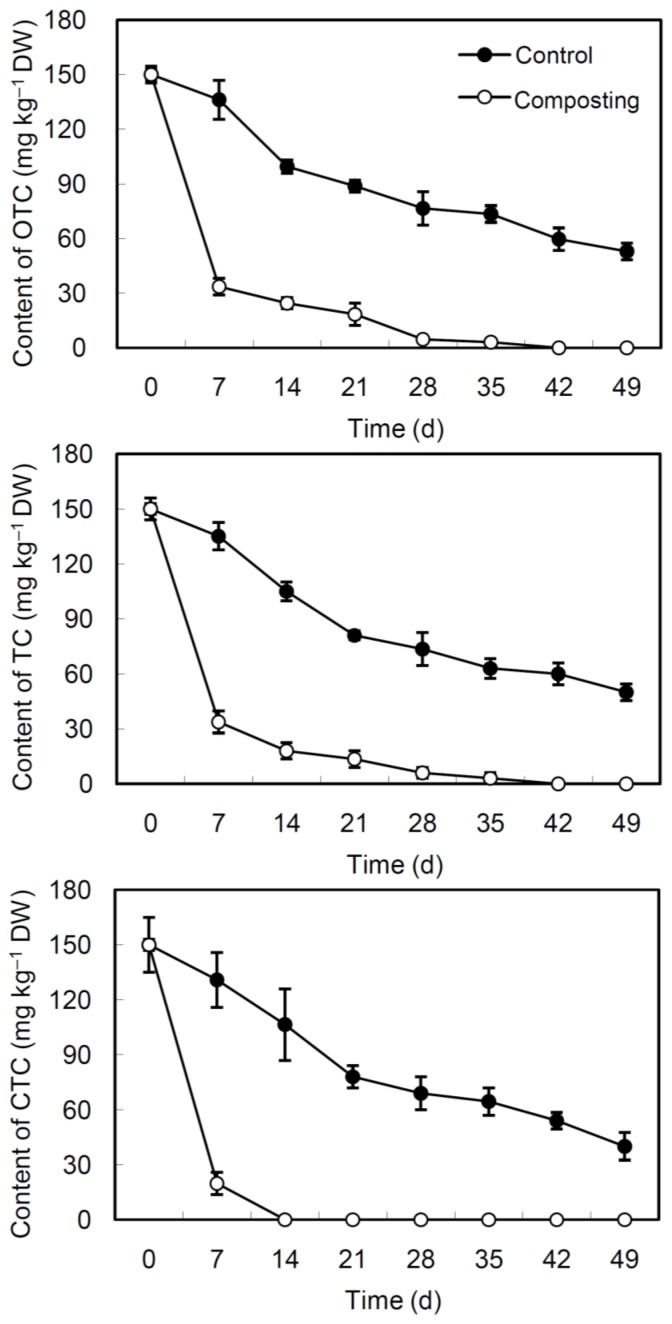
Degradation of tetracyclines during pig manure-rice straw composting and incubation at 25 °C in the dark (Control). OTC: oxytetracycline; TC: tetracycline; CTC: chlortetracycline. Bars represent standard deviation.

Degradation experiment of TCs showed that OTC, TC and CTC could be reduced rapidly during the composting process in greenhouse (Figure 3). During the first 7 days of composting, OTC, TC and CTC in the composting unit were degraded by 77.6%, 77.5% and 86.8%, respectively. While during the same period, the degradation of TCs in the control samples occurred slowly, with a removal rate of 9.2%, 9.9%, and 12.7% for OTC, TC and CTC, respectively. Complete removal of OTC, TC and CTC in the composting unit was achieved within 42 days, 42 days and 14 days, respectively. And this degradation behavior predominately took place in the thermophilic phase. However, at the end of the 49 days experiment, levels of OTC, TC and CTC in the control samples only decreased 64.7%, 66.7% and 73.3%, respectively.

The degradation of TCs during composting was modeled using the first-order kinetics: *C* = *C*_0_ · e^−*kt*^ [24,25,26], where *C* is the residue concentration (mg·kg^−1^) of TCs at time *t* (d), *C*_0_ is the initial concentration of TCs in the composting feedstock, and *k* is the degradation rate constant (d^−1^). Degradation time for 50% (DT_50_) of OTC, TC and CTC during composting were 5.5, 5.2 and 2.4 days, respectively. And degradation time for 90% (DT_90_) of TCs were 18.4, 17.4 and 7.9 d, respectively (Table 3). However, the DT_50_ (29.3, 27.1 and 26.9 days) and DT_90_ values (97.3, 90.1 and 89.5 days) of OTC, TC and CTC in the control samples were much longer.

## 4. Discussion

The present study clearly shows that pig manure-rice straw composting in a greenhouse for CO_2_ fertilization could be a powerful tool to accelerate the degradation of TCs in the composting feedstock. The TCs degradation depends on various environmental factors, including temperature, moisture and redox conditions as well as biological factors [27,28,29]. Temperature is an important factor that could influence TCs degradation [27]. In steer manure, OTC degradation was accelerated by increasing moisture and temperature under aerobic conditions, and thermal degradation became noticeable at high temperatures [28]. During the pig manure-rice straw composting process, the temperature in the composting pile was maintained at 50 to 65 °C for about 12 days. Therefore, high temperatures could significantly accelerate degradation of TCs. Yang *et al.* [29] found that the half-lives of OTC in soil under aerobic conditions were 29–56 days for non-sterile soil and 99–120 days for sterile soil. And in a recent study, it was discovered that microbial action is a major process that results in the degradation of TCs in swine wastewater [26]. In addition to elevated temperatures, rapid degradation of TCs during pig manure-rice straw composting in greenhouses could probably also be attributed to an intense biological activity. As reported in several investigations, composting has been identified as a feasible and effective way to reduce the environmental impact of antibiotics in manure [24,30,31,32]. Within the first six days of composting, levels of extractable OTC in beef manure mixed with straw and woodchips were reduced by 95% [33]. Due to temperature-dependent abiotic processes, concentrations of extractable CTC in beef manure mixed with straw and woodchips decreased rapidly, after composting at a temperature of 55 °C [25]. During pig manure composting, degradation of three tetracyclines CTC, OTC and TC predominately took place in the thermophilic stage (>50 °C) of the composting process [34]. Our data are consistent with these reports. In our experiment, complete removal of CTC in the composting unit was achieved within 14 days. And the degradation of OTC and TC was mainly completed in the first two weeks of composting. During pig manure-rice straw composting in greenhouses, the degradation rates of TCs in the composting feedstock were in the order of CTC > TC > OTC, and the half-life of CTC was 2.4 days. Similar results were reported by other researchers [24,25]. When composting at 55 °C, the half-life value for CTC in beef manure mixed with straw and woodchips was 4 days [25], while in spiked turkey litter, CTC concentration declined rapidly and the half-life for CTC was 1 day [24].

In China, increasingly high levels of synthetic fertilizer N are applied to croplands, especially for greenhouses. Serious concerns have been raised about the impacts of synthetic fertilizer N production on greenhouse gas emissions [35,36,37]. Therefore, seeking supplemental sources of nutrients and reducing synthetic fertilizers consumption is absolutely necessary. The application of composted residues from pig manure-rice straw composting could provide nutrients for vegetables and consequently, to some extent, reduce synthetic fertilizer consumption and thus reduce greenhouse gases emission in China.

## 5. Conclusions

Our study showed that the degradation of TCs was accelerated during pig manure-rice straw composting in greenhouses, thus decreasing the potential environmental risk of TCs-contaminated pig manure and making composted residues safer for field application. This composting procedure has a low implementation cost and is easy to operate. In addition, remarkable economic returns from increased vegetable yields should arouse the enthusiasm of greenhouse farmers for pig manure and rice straw collection and composting. It is expected that utilization of pig manure and rice straw for CO_2_ fertilization in combination with the application of TCs-free composted residues for greenhouse vegetables, would be readily accepted by greenhouse farmers.

## Figures and Tables

**Table 1:** Acid dissociation constant (p*K*a) values of tetracyclines.

Tetracyclines	p*K*_a1_	p*K*_a2_	p*K*_a3_
Oxytetracycline	3.27	7.32	9.11
Tetracycline	3.30	7.68	9.69
Chlortetracycline	3.30	7.44	9.27

**Table 2:** Characteristics of composting raw materials.

Characteristics	Materials		
	Pig Manure	Rice Straw	Mixture
Moisture content (%)	72.9	8.0	10.7
Organic carbon (g·kg^−1^ DW)	347	406	403
Total nitrogen (g·kg^−1^ DW)	25.3	9.4	10.1
C:N	13.7	43.2	39.8

**Table 3:** Degradation time (in days) for 50% (DT_50_) and 90% (DT_90_) of tetracyclines during pig manure-rice straw composting and incubation at 25 °C in the dark (Control).

Tetracyclines	Pig Manure-Rice Straw Composting		Control	
	DT_50_	DT_90_	DT_50_	DT_90_
Oxytetracycline	5.5	18.4	29.3	97.3
Tetracycline	5.2	17.4	27.1	90.1
Chlortetracycline	2.4	7.9	26.9	89.5
